# Diet modulates cecum bacterial diversity and physiological phenotypes across the BXD mouse genetic reference population

**DOI:** 10.1371/journal.pone.0224100

**Published:** 2019-10-21

**Authors:** Maria Elisa Perez-Munoz, Autumn M. McKnite, Evan G. Williams, Johan Auwerx, Robert W. Williams, Daniel A. Peterson, Daniel C. Ciobanu

**Affiliations:** 1 Department of Pathology, John Hopkins University School of Medicine, Baltimore, Maryland, United States of America; 2 Department of Agriculture, Food and Nutritional Sciences, University of Alberta, Edmonton, Alberta, Canada; 3 Animal Science Department, University of Nebraska, Lincoln, Nebraska, United States of America; 4 Laboratory for Integrative Systems Physiology, Ecole Polytechnique Fédérale de Lausanne, Switzerland; 5 Department of Genetics, Genomics and Informatics, University of Tennessee Health Science Center, Memphis, Tennessee, United States of America; University of Illinois at Urbana-Champaign, UNITED STATES

## Abstract

The BXD family has become one of the preeminent genetic reference populations to understand the genetic and environmental control of phenotypic variation. Here we evaluate the responses to different levels of fat in the diet using both chow diet (CD, 13–18% fat) and a high-fat diet (HFD, 45–60% fat). We studied cohorts of BXD strains, both inbred parents C57BL/6J and DBA/2J (commonly known as B6 and D2, respectively), as well as B6D2 and D2B6 reciprocal F1 hybrids. The comparative impact of genetic and dietary factors was analyzed by profiling a range of phenotypes, most prominently their cecum bacterial composition. The parents of the BXDs and F1 hybrids express limited differences in terms of weight and body fat gain on CD. In contrast, the strain differences on HFD are substantial for percent body fat, with DBA/2J accumulating 12.5% more fat than C57BL/6J (P < 0.0001). The F1 hybrids born to DBA/2J dams (D2B6F1) have 10.6% more body fat (P < 0.001) than those born to C57BL/6J dams. Sequence analysis of the cecum microbiota reveals important differences in bacterial composition among BXD family members with a substantial shift in composition caused by HFD. Relative to CD, the HFD induces a decline in diversity at the phylum level with a substantial increase in Firmicutes (+13.8%) and a reduction in Actinobacteria (-7.9%). In the majority of BXD strains, the HFD also increases cecal sIgA (P < 0.0001)—an important component of the adaptive immunity response against microbial pathogens. Host genetics modulates variation in cecum bacterial composition at the genus level in CD, with significant quantitative trait loci (QTLs) for *Oscillibacter* mapped to Chr 3 (18.7–19.2 Mb, LRS = 21.4) and for *Bifidobacterium* mapped to Chr 6 (89.21–89.37 Mb, LRS = 19.4). Introduction of HFD served as an environmental suppressor of these QTLs due to a reduction in the contribution of both genera (P < 0.001). Relations among liver metabolites and cecum bacterial composition were predominant in CD cohort, but these correlations do not persist following the shift to HFD. Overall, these findings demonstrate the important impact of environmental/dietary manipulation on the relationships between host genetics, gastrointestinal bacterial composition, immunological parameters, and metabolites—knowledge that will help in the understanding of the causal sources of metabolic disorders.

## Introduction

Substantial advances in omics technologies provided an abundance of key data that can be used to deconstruct the complex layers of causality between genetic, epigenetic, environmental, and phenotypic differences. For example, high-density genotyping, deep genomic and transcriptomic profiling led to deep characterization of mammalian and microbial genomes, dissection of the genetic variation of complex traits and interactions between host, environmental factors and pathogens. More recently, progress in the accuracy of mass spectrometry provided opportunities to generate relevant proteomic and metabolomic data to assemble coherent multilayered information across highly-characterized genetic reference populations (GRPs) [1].

In the last decade, sequencing of the gut microbiome provided a detailed picture of the diversity of the gastrointestinal bacterial communities (here referred as microbiota) with some ability to link roles of specific taxa on host metabolic phenotypes. The gastrointestinal system harbors a diverse microbiota with important roles in host metabolism. This microbial community is established early in life, influenced by maternal and environment factors and able to impact the health of the host [2]. For example, early studies provided evidence that diet plays an important role in the composition of gastrointestinal microbiota. Specifically, transition to a low-fat diet in overweight humans led to a gut microbial composition similar to that of healthy controls [3, 4]. Also, gnotobiotic animals displayed substantial weight gains following exposure to a complex gastrointestinal microbiota from overweight individuals [5, 6].

In the last decades mouse models have been extensively used to understand and validate the role of host genetics in variation of complex and diverse phenotypes important for human health. The purpose of this study is to evaluate host genetic responses and cecum microbial profiles to different levels of fat in the diet and to explore the potential impact of the gut microbiota on liver metabolites using a highly characterized mouse resource population, the BXDs. We achieved this by combining deep sequencing of cecum microbiota, genome-wide associations of the metagenomics data and an extensive analysis of the relationship between microbiota and liver metabolites in the BXD reference population subjected to different levels of fat in the diet, chow (CD, 13–18% fat) and high-fat diet (HFD, 45–60% fat).

The BXD family was derived from crosses of C57BL/6J and DBA/2J inbred strains. Family members are highly diverse, segregating for over 6 million sequence variants, but each BXD strain is fully inbred. The BXD strains differ markedly in their susceptibility to obesity, responses to dietary fat, longevity, as well as in thousands of behavioral, morphological and immunological traits. Differences among BXD strains are mainly explained by the random segregation of polymorphisms inherited from the two parents [7, 8]. A significant strength of the BXD is the high mapping power of a typical genetic resource family but also the ability to replicate any strain/genome many times and under different environmental and dietary conditions. This ability to resample any given genome many times provides efficiency in detecting causal pathways/networks across multiple environments and layers of phenome and transcriptome data—from gut microbiota to metabolic and immunologic traits [1, 7, 8, 9, 10, 11]. In this study we integrated multiple layers of phenotypic and genetic information, that span from changes in the gut microbiota to metabolic and immunologic traits, to analyze sources of the variation in response to high-fat diet and susceptibility to obesity.

## Results

### DBA/2J have higher susceptibility to obesity when subjected to HFD

**Fig 1:**
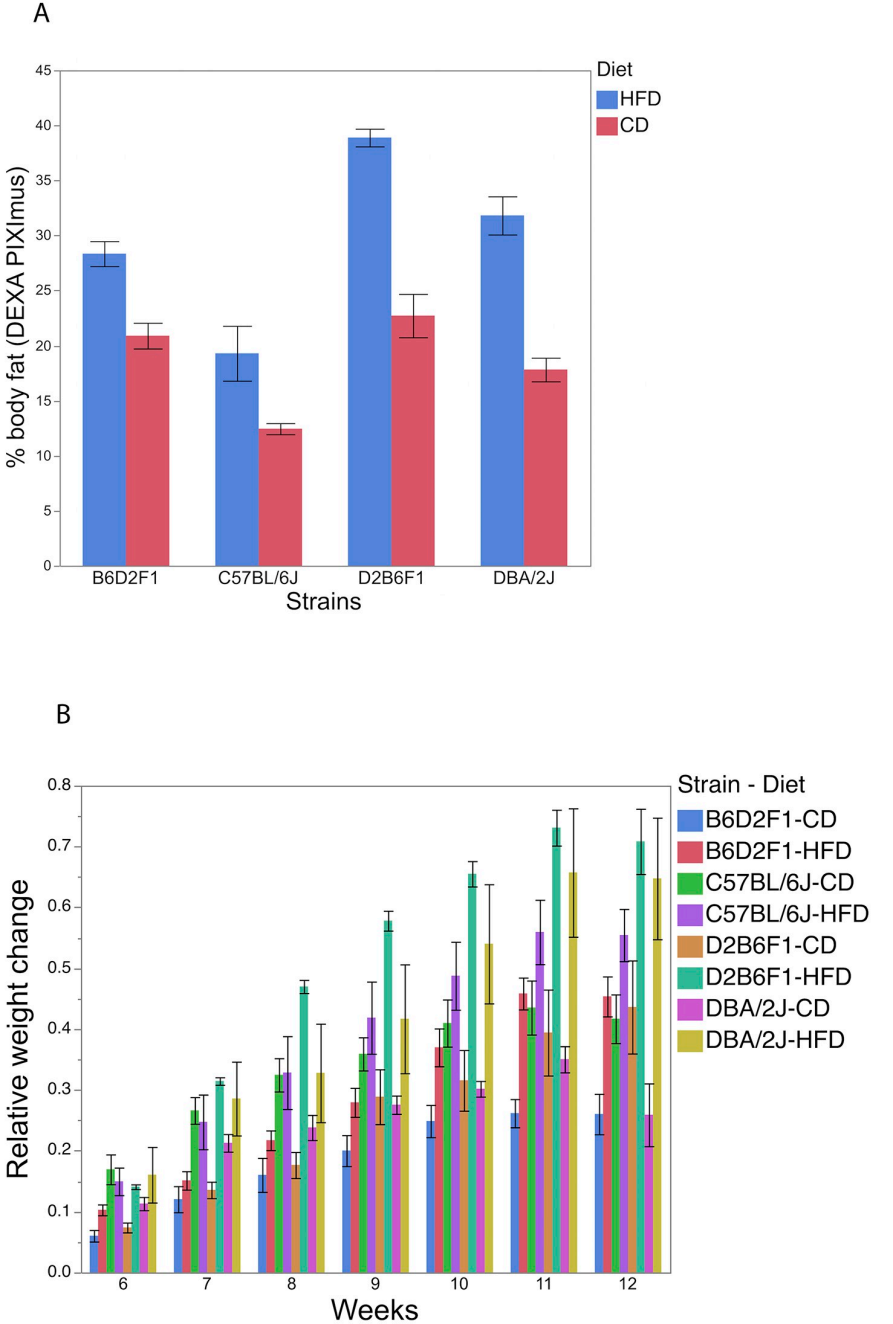
Proportion of body fat composition (12 weeks of age)(A) and changes in average body weight (g) relative to the start of dietary experiment (5 weeks of age)(B) in C57BL/6J, DBA/2J and their reciprocal F1 hybrids strains subjected to CD and HFD.

Due to important phenotypic differences between parental lines, C57BL/6J and DBA/2J, the BXD RI resource population has been used recently as a model for understanding the genetic control of a range of metabolic phenotypes [9] including mitochondrial functions [1, 11, 12]. In a study of the genetic response to levels of fat in the diet (CD vs HFD), a strain x diet interaction was observed for % body fat at 12 wks of age when C57BL/6J, DBA/2J and reciprocal F1 hybrids were subjected to 8 wks of dietary treatment starting at 5 weeks of age. No difference was observed between parental lines or between reciprocal hybrids (P > 0.20) when subjected to CD, while important differences were detected between strains when subjected to HFD (P < 0.05). For example, when subjected to HFD, DBA/2J had 12.5% more body fat compared to C57BL/6J (P < 0.0001, Fig 1A). Additionally, the F1 offspring generated by DBA/2J dams (DBA/2J x C57BL/6J) had 10.6% more body fat (P < 0.001) compared to the F1 from C57BL/2J dams (C57BL/6J x DBA/2J). While the source of these latter effects appears to be maternal, further studies are needed to identify the molecular basis of these differences. In general, genetic differences between strains impacted body weight variation throughout the experiment (P < 0.05) (Fig 1B). The diet had a significant effect on body weight starting at 2 weeks of dietary treatment (P < 0.05). All strains subjected to HFD expressed larger weight gain than those subjected to CD. The genetic response to dietary treatment was less evident on the body weight; a strain x diet interaction was found significant at 3 weeks of dietary treatment (P < 0.05) and suggestive at 2, 4–7 weeks of dietary treatment (P < 0.10).

### BXD parental strains have unique cecum microbial profiles

**Fig 2:**
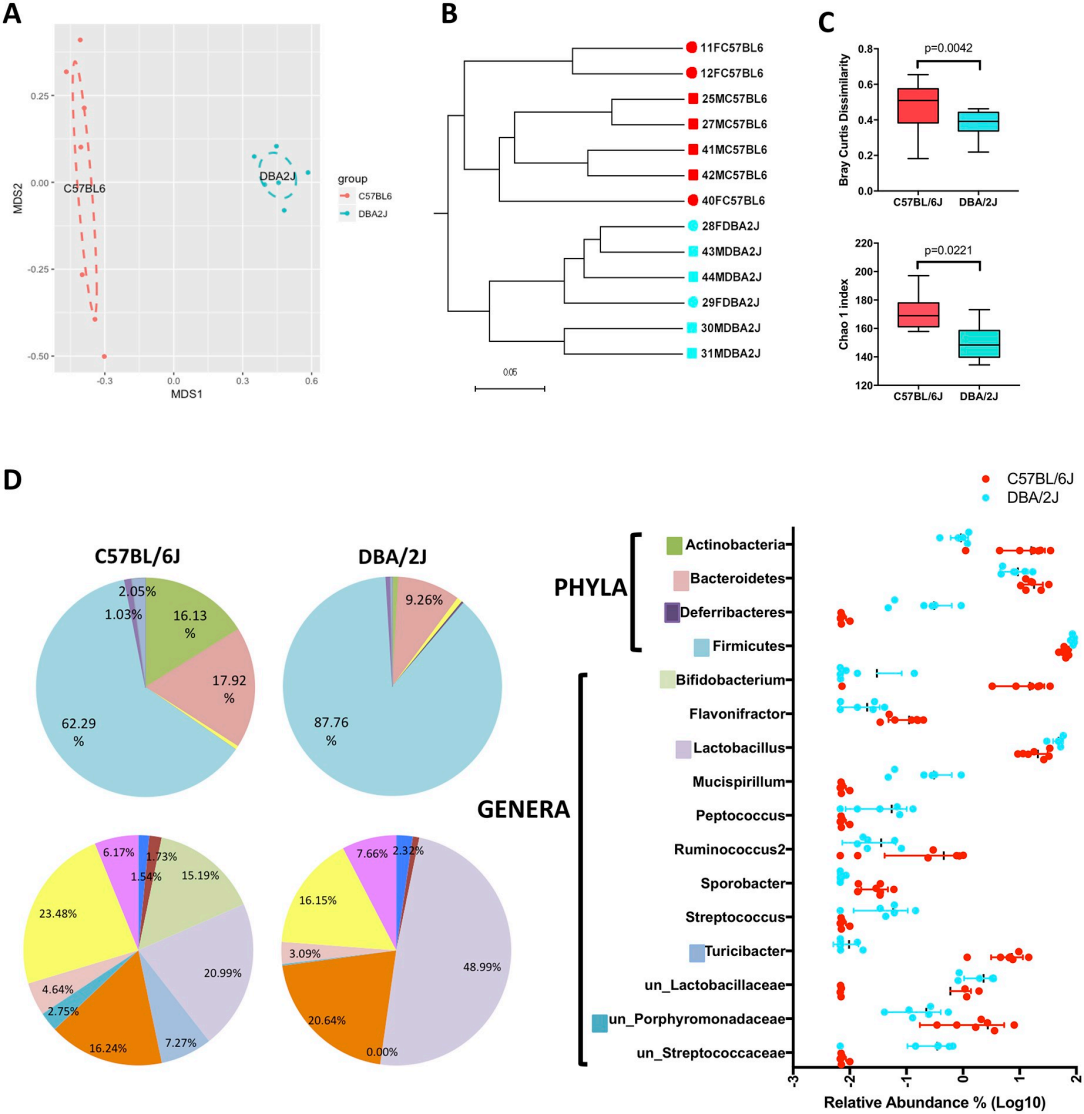
Gut microbiome composition of parental strains. Clustering of samples by NMDS of Bray Curtis dissimilarity (A) and dendrogram of Unweighted Unifrac distances (B) show that microbial communities of C57BL/6J mice are different from those of DBA/2J mice. Analysis of Bray Curtis dissimilarity (C-top) as a measure of β-diversity shows that DBA/2J mice have a lower degree of inter-individuality, while α-diversity analysis of Chao 1 index (C-bottom) shows that the gut microbiomes of C57BL/6J mice are richer and contain a higher number or rare species than those of DBA/2J mice. Pie charts and graph show relative abundance (D) of taxa present in both strains at the phylum, family and genus level. Taxa whose abundance was lower than 1% were grouped in the pie chart. Only statistically significant taxa are shown in graph on right. Statistics: Mann-Whitney t-test or Multiple t-test, lines and bars represent mean and standard deviation.

Our study of cecum microbiota indicates complete dissimilarity of the microbial communities between C57BL/6J and DBA/2J strains (Fig 2A and 2B). DBA/2J mice have a lower degree of inter-individuality (P = 0.0042; Fig 2C-top) while the gut microbiomes of C57BL/6J mice are richer and contain a larger number or rare species than those of DBA/2J mice (P = 0.0221, Fig 2C-bottom) (S1 Table). The gut microbiota of C57BL/6J and DBA/2J was dominated by Firmicutes, contributing with 62% and 88%, respectively (Fig 2D). Actinobacteria and Bacteroidetes were less represented in DBA/2J, accounting for 9% and 1%, respectively, compared to C57BL/6J, where these phyla were represented by 18% and 16%, respectively. At the genera level, *Lactobacillus* was the most abundant genus in both strains, although higher in DBA/2J than C57BL/6J (49% and 21%, respectively). *Bifidobacterium* and *Turicibacter* are the following two most abundant genera in C57BL/6J (15% and 7%), while their abundance in DBA/2J was marginal (< 1%).

The effect of sex and maternal cage were partially confounded and could not be directly estimated. When included as a combined effect in the model (as contemporary group) together with the strain, the differences between strains reported above were still observed. In addition, combined effects of the sex/maternal cage were observed in Clostridia, Bacilli, Bacteroidia and Deltaproteobacteria classes (P < 0.05).

### Cecum microbial profile of the BXD strains is influenced by dietary change

**Fig 3:**
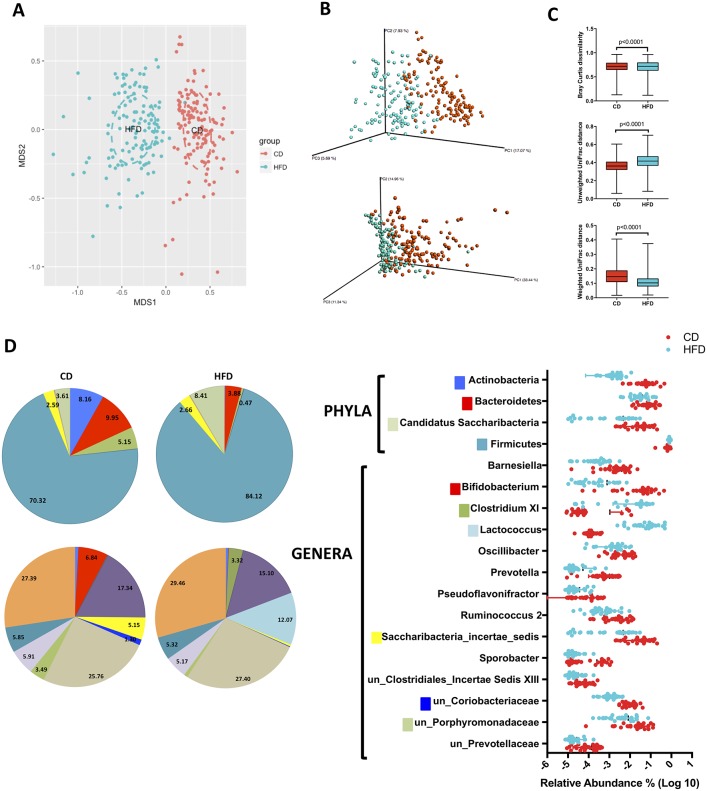
High fat diet causes shifts in the gut microbiomes of BXD. Clustering of samples by NMDS of Bray Curtis dissimilarity (A) and principal coordinates analysis (PCoA) of Unweighted and Weighted Unifrac distances (B) show a shift in the microbial communities of the gut in the BXD mice caused by the HFD. Analysis of β-diversity metrics show statistically significant differences between the dietary treatments (C). Pie charts and graph show relative abundance (D) of taxa present in both strains at the phylum and genus level. Taxa whose abundance was lower than 1% were grouped in the pie chart. Only statistically significant taxa (p<0.05) are shown in graph on right. un_: unclassified Statistics: Multiple t-test, lines and bars represent mean and standard deviation.

Substantial dissimilarity and a shift in the microbial communities were observed across BXD strains subjected to HFD relative to the CD (Fig 3C). There was a limited overlap in abundance between the BXD subjected to both diets in genera that exhibited significant changes triggered by HFD with the exception of *Lactococcus*, where there is complete separation between the BXD exposed to the different diets (Fig 3). Cecum microbial profile was dominated by Firmicutes in both CD and HFD contributing with 70.3% and 84.1%, respectively. Bacteroidetes were the second most represented phylum (9.6%) in the CD group, followed by a similar contribution from Actinobacteria (8.2%). Introduction of HFD triggered significant changes in cecum microbial profile in four taxa at the phylum level (P < 0.006) and 14 genera (P < 0.0007). At the phylum level, HFD induced a reduction in microbial diversity with major changes represented by a substantial increase in Firmicutes (+13.8%) and a reduction in Actinobacteria to a marginal representation (-7.9%). Major changes at the genus level driven by HFD include a substantial increase in *Lactococcus* (+12.1%), a member of Firmicutes, and a reduction in *Bifidobacterium* (-6.8%), a member of Actinobaceria (S2 Table). As expected, HFD causes some taxa to be non-detectable relative to CD.

### HFD induced an increase in cecal sIgA, with a response that varied across BXD strains

**Fig 4:**
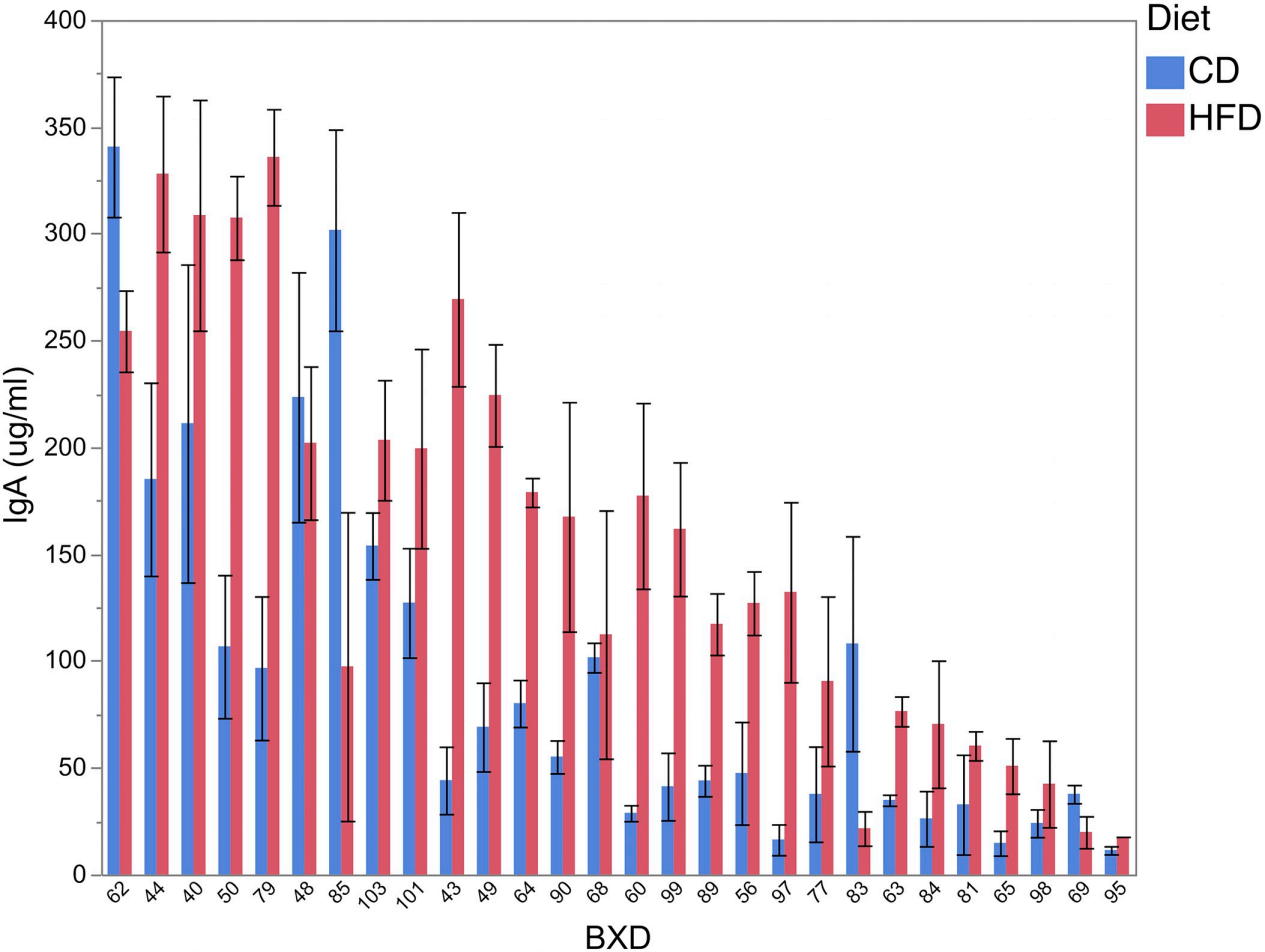
Cecal sIgA in BXD strains subjected to CD and HFD. HFD induced an increase in cecal sIgA, with a response that varied across BXD strains.

Secretory IgA (sIgA) is known not only as an important component of adaptive immunity against microbial pathogens [13, 14], but it can also influence composition of the gut microbiota [15]. Introduction of HFD led to a significant increase in cecal sIgA compared to CD (P < 0.0001, Fig 4). With the exception of few strains (BXD48, BXD62, BXD69, BXD83 and BXD85), the majority of strains (82.1%) experienced a nominal increase in cecal sIgA in HFD, with 35.7% of them experiencing a significant increase (P < 0.05). The largest increase in sIgA was observed in BXD97 (+80.4%). The largest reduction in sIgA was observed in BXD83 (-34.3%).

The role of sIgA in the modulation of microbial composition was assessed across taxa and dietary treatments. For example, it would be important to know if variation in sIgA could impact gut microbiota in BXD subjected to CD but also following introduction to HFD. The largest relationship between sIgA and cecum microbiota in CD was observed for *Sporobacter* (r = -0.45, P < 0.05). The relative abundance of this genus, a member of Ruminococcaceae (Firmicutes), was found decreased in HFD (P = 0.01). On HFD, the largest relationship was observed between sIgA and *Verrucomicrobia* (r = -0.38, P < 0.05).

In order to assess the role of host genetics in explaining the observed differences in sIgA, Quantitative Trait Locus (QTL) mapping was employed using a set of 3,785 DNA markers genotyped across the BXD strains. In the CD cohort a suggestive QTL (P = 0.08) was detected on Chr 4 (22.6–23.3 Mb) that accounts for 6.7% of the phenotypic variance in sIgA, with C57BL/6J allele increasing the trait. Gene ontology analysis of the QTL region and a review of transcript expression in the gastrointestinal tract, did not indicate any strong candidate genes linked to sIgA variation.

### Host genetics influences microbial composition of mouse cecum

QTL analysis of cecum microbiota composition from phylum to genus in 29 weeks-old BXD strains revealed two significant QTLs (P <0.05) for *Oscillibacter* (Chr 3, 18.7–19.2 Mb, LRS = 21.4, S1 Fig) and *Bifidobacterium* (Chr 6, 89.2–89.4 Mb, LRS = 19.4), both observed in the CD cohort (S3 Table). The introduction of HFD overwrote any potential implication of the host genome on microbial profile, since these QTLs were absent in BXD subjected to HFD. Proportion of the phenotypic variance explained by these QTLs was 5.6% for *Oscillibacter* and 11.3% for *Bifidobacterium*. The abundance of *Oscillibacter* in BXD cecum was increased by DBA/2J allele while the abundance of *Bifidobacterium* was increased by C57BL/6J allele. Introduction of HFD led to a reduction in the contribution of these genera in cecum microbiota (P < 0.001). For example, *Bifidobacterium* contribution decreased drastically from 6.84% in CD to a marginal contribution of 0.08% in HFD (P < 0.0001). *Bifidobacterium* was the main contributor of the Actinobacteria phylum in CD (83.8%) and the reduction of its presence was a source of the decrease in Actinobacteria (P < 0.0001) in HFD (0.23%) compared to CD (8.16%). A reduction in *Oscillibacter*, a member of Firmicutes, was also observed in HFD (P <0.001) while the proportion of Firmicutes increased in BXD subjected to HFD compared to CD (P < 0.0001).

The impact of host genotype on the microbial composition was also evaluated at the species level using Operational Taxonomic Unit (OTU) data. QTLs involving 24 OTUs were mapped on 13 chromosomes (P < 0.05). The QTL presence was split almost equally between dietary treatments, CD (12 QTLs) and HFD (13 QTLs). The OTU171 (*Lachnospiraceae*) was the only species with QTLs identified in both CD (Chr 10) and HFD (Chr 5) but on different chromosomes. The majority of the QTLs identified at species level were associated with *Lachnospiraceae* (9 QTLs) followed by *Ruminococcaceae* family (3 QTLs). The contribution of each QTL to the phenotypic variation of these species (OTUs) varied from 4.5% (OTU422, *Adlercreutzia* genus) to 17.4% (OTU94, *Lachnospiraceae* family).

The QTLs detected for *Oscillibacter* and *Bifidobacterium* genera were also identified at the species level. Specifically, a QTL associated with *Bifidobacterium pseudolongum* (OTU4) was detected at the same position as for genus *Bifidobacterium* (Chr 6, 89.2–89.4 Mb). Similarly, a QTL located at the same position (Chr 3, 18.7–19.2 Mb) was detected for both *Oscillibacter* genus and its species, *Oscillospira* (OTU17). The direction of the QTLs effect at the species level was the same as detected at the genus level. The abundance of *Oscillospira* in BXD cecum was increased by DBA/2J allele while the abundance of *Bifidobacterium pseudolongum* was increased by C57BL/6J allele.

Sequence data in the QTL regions and gene expression in the gastrointestinal tract were analyzed to identify candidate genes that could explain some of the variation in *Bifidobacterium pseudolongum* and *Oscillospira* levels. There was no evidence of *cis*-modulated mechanisms of the transcripts in the gastrointestinal tract within both QTLs (Chr 3, 18.5–20; Chr 6, 89–90 Mb). Polymorphisms potentially affecting splicing were identified in candidate genes located on both QTL regions. Phosphodiesterase 7A (*Pde7a*) is one of the candidate genes located in the QTL interval of Chr 3 and associated with *Oscillospira*. *Pde7a* is characterized by alternative splicing variants; the SNP associated with the largest QTL effect for *Oscillospira* (*rs31338665*, Chr 3, LRS = 19.6, P < 0.05) is located within the fifth intron of *Pde7a* and only 1,455 bp away from *rs30549686*, a splice region variant located in intron 4 and a candidate for the detected QTL. One of the candidate genes located in the QTL interval of Chr 6 and associated with *Bifidobacterium pseudolongum* was thioredoxin reductase 3 (*Txnrd3*). *Txnrd3* is expressed in the gastrointestinal tract and is characterized by limited genetic variation in BXD. However, an intronic SNP located (*rs13478882*) next to an alternatively spliced exon (exon 8) represents a candidate for the observed phenotype. This SNP is located 248 kb from the QTL SNP associated with the largest effect (*rs30220244*).

### Relations between liver metabolites and cecum microbiota

BXD and their parental strains, C57BL/6J and DBA/2J differ for a range of developmental and physiological traits [10, 12]. For example, BXD strains display substantial differences for indicator traits of metabolic syndrome such as liver weight, serum cholesterol, fasted glucose and liver metabolites in both CD and HFD [12]. To examine the role of cecum microbiota in susceptibility to metabolic syndrome, the relationship between cecum microbial composition and a large set of liver metabolites [1]. In this analysis we included 699 metabolite features mapped to a specific metabolite based on Human Metabolome Database (HMDB) annotations and 280 metabolites mapped to multiple possible enantiomers, as described [1].

**Fig 5:**
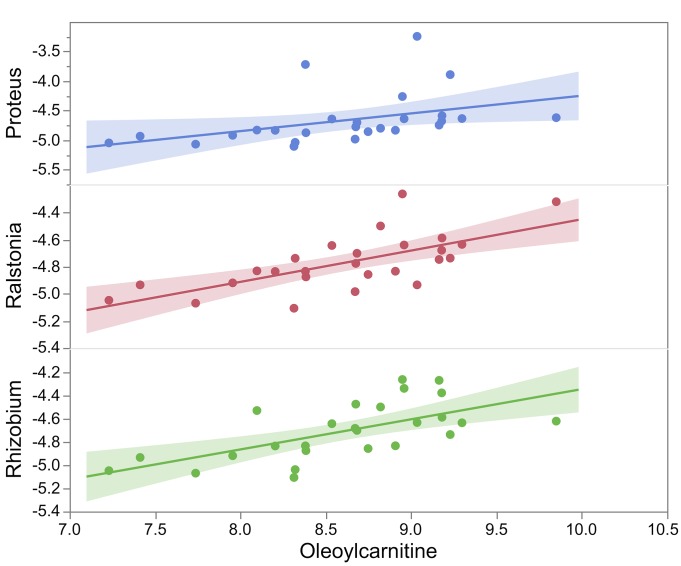
Phenotypic relationships between oleoylcarnitine, a liver metabolite linked to uremic cardiovascular risk, and abundance of three members of Proteobacteria in BXD (*Proteus*, *Ralstonia* and *Rhizobioum*, r>0.67, P≤0.0001).

Phenotypic relations were evaluated between the taxonomic units at the phyla and genus level and the liver metabolites measured in 40 BXD strains subjected to CD and HFD. Significant correlations (P ≤ 0.0001) were observed in the CD group between taxonomic units across phyla and the liver metabolites. Three members of Proteobacteria (*Proteus*, *Ralstonia* and *Rhizobioum*, r > 0.67, Fig 5) were correlated with oleoylcarnitine level, a marker of uremic cardiovascular risk [16]. *Brevundimonas*, another Proteobacteria, was positively correlated with caprylic acid, involved in fatty acid biosynthesis (r = 0.72). *Oscillibacter*, (Firmicutes) a member of Ruminococcaceae family, was positively associated with metabolites associated with glycerophospholipid (e.g. phosphatidylserine, phosphatidylethanolamine) and glycerolipid metabolism (trygliceride) (r > 0.68). *Sporobacter* (Ruminococcaceae) was negatively associated with dihydroneopterin phosphate involved in folate biosynthesis (r = -0.69). Other Firmicutes were associated with metabolites related to tryptophan metabolism (4,6-Dihydroxyquinoline, r = 0.70) such as *Blautia*, or with cholesterol metabolism (cholesterol ester, r = -0.68) such as *Weissela*. The only observed relationship for Bacteroidetes was found between *Prevotella* and a metabolite associated with the glycerophospholipid metabolism (lysophosphatidylethanolamine, r = 0.70).

In HFD, significant relations (P ≤ 0.0001) between taxonomic units and the liver metabolites were observed across phyla, in Firmicutes, Bacteroidetes and Proteobacteria. For example, the abundance of Firmicutes was negatively associated with level of troxerutin (r = -0.70), a flavonoid known for potential antioxidant properties. *Lactococcus*, a member of Firmicutes, was associated with level of crotonoyl-CoA (r = 0.72), an important component of several metabolic pathways including fatty-acid metabolism. Negative relationships were observed between *Parabacteroides*, a genus belonging to Bacteroidetes, and molybdopterin-AMP (r = -0.73) and positive relationships (r = 0.72) with carbamoyl phosphate. Lastly, *Biophila*, a genus of Proteobacteria, was associated with p-cresol glucuronide (r = 0.71), a metabolite resulting from tyrosine biotransformation by anaerobic intestinal bacteria.

## Discussions

This study provides additional evidence of important phenotypic and genetic variation for traits associated with metabolic syndrome in two of the most important mouse genetic lines, C57BL/6J and DBA/2J and their related BXD RI resource population. Introduction of HFD captured differences between BXD parental lines and F1 hybrids not seen in control CD. Specifically, DBA/2J and F1 hybrids born to DBA/2J dams accumulated more body fat compared to C57BL/6J and F1 hybrids born to C57BL/6J dams when subjected to HFD, indicating a higher susceptibility to obesity in DBA/2J.

We also found that BXD parental strains have unique cecum microbial profiles. At the phylum level, while both strains were dominated by Firmicutes, C57BL/6J is characterized by larger diversity having important contributions of the other major phyla, Actinobacteria and Bacteroidetes. While some of the differences in microbial profile between parental strains were potentially confounded with the maternal cage effects, clustering of all C57BL/6J samples generated by 3 different litters in the same phylogenetic group suggest a role of host genetics in the modulation of cecum microbial profile (Fig 2A and 2B). Moreover, introduction of HFD in BXD served as an environmental suppressor, increasing the cecal sIgA levels, reducing the diversity of the cecum microbiota, disrupting the role of host genetics on the composition of the gut microbiota and also affecting the relationships between gut microbiota and liver metabolites.

IgA represents an important component of the adaptive immune response against microbial pathogens and could influence composition of gut microbial flora [15]. A recent report found that a short-term exposure of mice to HFD (2 wks) increased the abundance of Firmicutes and affected pro-inflammatory gene expression profile, increasing host susceptibility to *Listeria monocytogenes* infection [17].

Potential evidence of host genetics contribution to the variation in cecum microbiota was shown in CD for *Oscillibacter/Oscillospira* (mapped to Chr 3) and for *Bifidobacterium/Bifidobacterium pseudolongum* (mapped to Chr 6). Introduction of HFD in BXD served as an environmental suppressor of these QTLs, potentially due to a reduction in the contribution of these genera in BXD cecum. Potential genes associated with the QTL effects include *Pde7a* (Chr 3) and *Txnrd3* (Chr 6). Ontologies associated with *Pde7a* included metal ion binding. Essential metal ions are critical in important cellular processes of microbial species [18]. Co-expression analysis between *Pde7a* and known genes in modules/pathways based on two intestinal transcriptome datasets (GSE59054 and GSE6065, n>80) using Genebridge (www.systems-genetics.org/) revealed a suggested role for *Pde7a* being associated with *intestinal immune network* for IgA production (Score > 840; KEGG 04672). Considering the proposed role of *Pde7a* in modulating proportion of *Oscillospira*, we hypothesized a significant relationship between this species and sIgA. As expected, the level of sIgA was correlated with *Oscillospira* in CD (r = -0.41, P<0.05) but not in HFD (P>0.14), corroborating the suggested role of *Pde7a* in co-expression networks associated with intestinal IgA production.

*Txnrd3*, the potential candidate gene associated with the QTL for *Bifidobacterium pseudolongum* (*Bifidobacterium*), is a member of selenocysteine-containing proteins (selenoproteins) family playing an important role in redox homeostasis [19]. Expression of *Txnrd3* was reduced in mouse RAW264.7 macrophage cell line following lipopolysaccharide (LPS) treatment [20]. Selenium treatment rescued *Txnrd3* expression and reduced immunological stress associated with LPS. Dietary selenium intake affects expression of host selenoproteins but also the diversity of the gut microbiota [21]. Gut microbiota extracts selenium from diet. In cases of limited selenium intake, competition between microbiota and host could occur. This condition could lead to decreased expression of selenoproteins and antioxidative activity [22]. *Bifidobacteria* are able to accumulate and biotransform inorganic selenium providing a source of organic selenium to the host.

Relations among liver metabolites and cecum bacterial composition were predominant in CD and did not persist in HFD. The relationships between intestine, microbial communities and liver are well documented [23, 24, 25]. The communication maintained via portal vein and biliary system, expose the liver to metabolites and microbial products derived from intestine. For example, the bile acids are synthesized in the liver from cholesterol and later metabolized by the intestinal microbiota into secondary bile acids, conversion that influence signaling properties of bile acids regulating host metabolic pathways. Any changes that affect the function of the intestine as a barrier, including a diet rich in saturated fats that elevate levels of resident bacteria with pathogenic potential, can lead to leakage of bacterial metabolites and liver and metabolic disease. Significant changes in liver metabolites due to HFD were the potential cause of the loss in the significant relationship with gut microbiota, as observed in CD. For example, there was a significant reduction in the metabolites associated with the glycerophospholipid metabolism in HFD compared to CD (e.g. phosphatidylserine, phosphatidylethanolamine, lysophosphatidylethanolamine). Some of the relationships detected in HFD were potentially driven by changes in microbiota profile, as in the case of *Lactococcus*, which resulted in an increase in abundance in HFD compared to CD, since level of crotonoyl-CoA in the liver did not change between diets.

The general increase in cecal sIgA in HFD was not associated with any changes in liver metabolites. However, a negative relationship was observed in CD between the level of sIgA and galactosylsphingosine (r = -0.73), a compound associated with sphingolipid metabolism. Sphingolipids are important factors in modulating sIgA responses [26]. Dietary palmitic acid is metabolized into sphingolipids such as sphingosine (also a substrate of galactosylsphingosine), which is absorbed into intestinal tissue and is subsequently metabolized into sphingosine 1-phospate, known to modulate cell-trafficking and sIgA responses to oral antigens.

In conclusion the results of this study demonstrate the important role of environmental/dietary manipulation on the interactions between host genetics, immunological parameters and gastrointestinal microbiota and their impact on metabolites that could serve as indicators of health status. Deep understanding of these fine relationships can provide the knowledge that could help deconstruct the causal sources of metabolic disorders.

## Materials and methods

### C57BL/6J, DBA/2J and reciprocal F1 dietary experiment

In order to determine the baseline diversity of the cecal microbiome in the parental strains of BXD, cecum samples were collected from naive males and females from C57BL/6J and DBA/2J with an average age of 11 weeks. The experimental mice originated from three (C57BL/6J, n = 7, average age 72 d) to four (DBA/2J, n = 6, average age 87 d) litters. The mice were housed in a specific pathogen-free environment at 20–24°C with a light/dark cycle of 14/10 hr with *ad libitum* access to water and food at the University of Tennessee Health Science Center (UTHSC). Male and female littermates were housed in separate cages. The mice were subjected to a normal chow diet (CD, Harlan Teklan 22/5; 17% calories from fat, 54% calories from carbohydrate, 29% calories from protein). All animals were kept in accordance with guidelines set by the NIH Guide for the Care and Use of Laboratory Animals and under the prevue of the Institutional Animal Care and Use Committee (IACUC) at the UTHSC. The IACUC at the UTHSC specifically approved the study (Permit no. 680).

The impact of high fat diet (HFD) on body weight and body composition was investigated by subjecting C57BL/6J, DBA/2J and reciprocal F1 hybrids to two dietary treatments for 8 weeks, starting at 5 weeks of age. This experiment included 4–8 male mice/strain/diet subjected either to a normal chow diet (CD, Harlan 7001; 13% calories from fat, 53% calories from carbohydrate, 34% calories from protein) or a high fat diet (HFD, Harlan TD.06415; 45% calories from fat, 36% calories from carbohydrate, 19% calories from protein). The mice were housed in a specific pathogen-free environment at 20–24°C with a light/dark cycle of 14/10 hr and *ad libitum* access to water and food at the University of Nebraska-Lincoln (UNL). Individual weight and cage food intake were recorded weekly starting from week 5 until week 12. Body composition including bone mineral density, proportion of lean and fat tissue, was recorded at 12 weeks on 4–5 mice/strain/diet using PIXImus DEXA. This study was approved by the UNL IACUC (Permit no. 422).

### BXD dietary experiment

Cecum was collected from 29 weeks old males of 32 BXD strains exposed to 2 diets, CD and HFD, including 3–5 animals/strain for each diet. There were 30 BXD strains represented in CD diet (Harlan 2018; 18% calories from fat, 58% calories from carbohydrate, 24% calories from protein) while 29 BXD strains were represented in the HFD diet (Harlan 06414; 60% calories from fat, 21% calories from carbohydrate, 18% calories from protein). HFD was introduced to HFD cohort starting from 8 weeks and maintained until tissue collection at 29 weeks. The mice representing each BXD strain and diet were housed in the same cage until week 23, followed by individual housing until tissues collection at 29 weeks after an overnight fast.

Euthanasia was performed using isoflurane anesthesia followed by a complete blood draw from the vena cava and perfusion with phosphate-buffered saline as described in Williams et al., (2016). Cecum, including cecum content, and liver tissue were frozen in liquid nitrogen followed by storage at –80°C for mRNA, protein, metabolite analysis and microbiota (for cecum content). The research protocols were approved by the Swiss Cantonal Veterinary Authorities of Vaud (licenses 2257.0 and 2257.1).

Liver metabolite profiles were acquired using time-of-flight mass spectrometry (ToF-MS) on an Agilent 6550 QTOF in negative mode at 4 GHz scanning for features between 50–1000 Da using the protocol as described by Fuhrer et al. (2011) [27].

There were 979 unique metabolite features being identified. Of these, 699 were mapped to a specific metabolite based on Human Metabolome Database (HMDB) annotations, while the remaining 280 mapped to multiple possible enantiomers, as described [1]. The data set is available in GeneNetwork.org database (EPFL/LISP BXD Liver Polar Metabolites CD / HFD, Jun 14).

### Next-generation Sequencing of Cecum-specific Microbial DNA

Sequencing of the cecum microbial DNA in parental strains was performed using samples collected from naive males and females from C57BL/6J (n = 7) and DBA/2J (n = 6) with an average age of 11 weeks (source: UTHSC, see above). Sequencing of the cecum microbial DNA was also performed using samples collected from 29 weeks-old males from 32 BXD strains exposed to CD and HFD (source: EPFL, see above; 3–5 animals/strain/diet). After three washes with ice-cold phosphate buffer saline (PBS), DNA was extracted from 100-150mg of cecal contents using the QIAmp DNA stool Mini Kit (Qiagen) following mechanical cell lysis as described previously [10]. The supernatant from the first wash, which was 10 times volume per weight of cecal contents, was stored at -80°C for sIgA measurements. Extracted DNA was initially amplified using universal primers for the V5-V6 region of the 16S rRNA gene and containing bar-coded adapters. The forward primer used was 784F (5’-RGGATTAGATACCC-3’) and the reverse primer was 1064R (5’-CGACRRCCATGCANCACCT-3’). Amplicon sequencing was performed using Illumina MiSeq pair—end chemistry at the University of Minnesota Genomics Center. Reads were trimmed to 240 bp with the FASTX-Toolkit (http://hannonlab.cshl.edu/fastx_toolkit/), and paired-end reads were merged with the merge-illumina-pairs application (https://github.com/meren/illumina-utils/) using the following criteria: (i) *P* value of 0.03, (ii) enforced Q30 check, (iii) perfect matching to primers, and (iv) no ambiguous nucleotides allowed. Standardizing the sequencing depth across samples was based on the lowest number of reads per sample, files being subsampled using Mothur v.1.35.1 to 10,000 sequences per sample for the diet comparison, and to 150,000 reads per sample for the strain comparison. Subsequently, USEARCH v8.1.1861 was used to generate operational taxonomic units (OTUs) with a 98% similarity cutoff. OTU generation included the removal of putative chimeras identified against the Gold reference database, in addition to the chimera removal inherent to the OTU clustering step in UPARSE. The resulting reads were assigned to different taxonomic levels from Phylum to Genus using a parallelized version of CLASSIFIER (rdp_classifier_v2.10.1) from the Ribosomal Database Project (RDP) as described in Benson et al. (2010) [28]. The number of reads in each taxonomic bin was normalized by using pseudocounts and calculating proportions of the total number of reads per each sample before statistical analyses. Diversity analyses were done using MacQIIME version 1.9.1.

### Cecum secretory Immunoglobulin A (sIgA) ELISA

A standard ELISA protocol was used for measurement of sIgA in the supernatants of previously washed cecal contents (cecal contents were washed three times in 10 times its weight with ice cold PBS before DNA extraction; the initial wash solution was used for sIgA measurement). Briefly, plates (NUNC MaxiSorp ELISA) were coated with 100μl of unlabeled goat anti-mouse (GAM) Ig heavy and light chain (H&L) (Southern Biotech #1010–01) diluted 1:1,500 in bicarbonate buffer and incubated for 2 hours at room temperature (RT). Coating buffer was decanted and after 3 washes with PBS-Tween (0.5% Tween 20 in PBS, pH 7.4, PBS-T) wells were blocked with 300ul 1% Bovine Serum Albumin in PBS for 30 minutes at RT. Subsequently, 100ul of serial dilutions of samples and standards were plated in duplicates. Plates were covered and incubated at RT for 2 hours. Unlabeled mouse IgA (Southern Biotech #0106–01, clone S-107) was used for the standard curve at an initial concentration of 500ng/ml. After incubation, plates were washed three times as previously, and 100μl of IgA-HRP (Southern Biotech #1040–05) diluted 1:1,500 were added to each well. Plates were incubated 2 hours at RT followed by three additional washes with PBS-T. After the last wash, 100μl of 2,2'-Azinobis [3-ethylbenzothiazoline-6-sulfonic acid]-diammonium salt substrate (ABTS) activated with fresh hydrogen peroxide (1μl/ml) were added to each well, and OD was read at 405nm.

### Statistical analysis

The effect of the dietary treatments on body weight and % body fat of BXD parental lines, C57BL/6J and DBA/2J, and their reciprocal F1 hybrids was tested using a linear mixed model in JMP Pro 13.1 using strain, diet and strain x diet as fixed effects and cage as random.

To achieve normality for the microbiome data that was not normally distributed, values were subjected to log 10 transformations. For comparisons between two groups (CD-HFD comparison), Student’s *t* tests with Welch’s correction were performed, while Kruskal-Wallis tests followed by Dunn’s multiple comparisons were applied for comparisons between more than two groups (strains samples). Statistical analyses were performed using Graph Pad Prism version 7 (GraphPad Software, La Jolla, CA, USA) and the statistical software R version 3.3.1 (https://www.r-project.org/).

Males and females representing the same parental strains (C57BL/6J and DBA/2J) did not originate from common maternal cages and within strain, the sex was not fully represented in multiple cages. Due to confounding effects the sex and maternal cage was combined as a contemporary group (CG) effect in a linear model (strain fixed effect and CG nested in strain).

The effect of diet on cecum microbial profile of BXD was also tested using a linear mix model, using diet as fixed effect and strain genetics as random. The effect of age and order of sacrifice was tested as covariates and found not significant when adjusted for multiple testing.

Phenotypic correlations between cecum bacterial composition at the phyla and genus level, as defined above, and the liver metabolites were estimated by Spearman rank correlations.

### QTL mapping

QTL mapping was based on the regression of the 3,785 polymorphic DNA markers on the targeted phenotypes, using QTL Reaper, as previously described [10]. The phenotypes used include the sIgA levels and the average proportion of each microbiota taxa. To achieve normality, taxonomic abundance was subjected to log 10 transformations, as described above. The marker genotypes were coded as -1, 0 and +1 to define C57BL/6J, heterozygote and DBA/2J genotypes. The threshold of the logarithm of the odds (LOD) score for genome-wide significant QTL (P < 0.05) was determined using empirical *P*-values from 5,000 permutations of the strain average phenotype (microbial profile) using GeneNetwork (GeneNetwork.org). Likelihood ratio statistic (LRS) scores were obtained by multiplying LOD by 4.61. Confidence intervals for the detected QTLs were defined by a LOD drop of 1.5 on either side of the peak LOD value.

## Supporting information

S1 Fig Genome-wide QTL mapping of *Oscillibacter* in the cecum of BXD strains. The Left y axis represents the Likelihood Ratio Statistic (LRS) between *Oscillibacter* composition to different DNA marker intervals on A) each chromosome (blue line) and B) detailed region of the QTL located on Chr 3 (18.7–19.2 Mb) that explains 5.6% of the composition in *Oscillibacter*. The Right y axis represents the additive effect and indicates if the DBA/2J (green line) or C57BL/6J (red line) alleles contributes to an increase in the abundance of *Oscillibacter*. Pink and gray horizontal lines indicate the significant (<0.05) and suggestive (<0.67) QTL threshold.
(TIFF)

S1 Table Gut microbiome composition of parental strains of BXD, C57BL/6J and DBA/2J. (TXT)

S2 Table Gut microbiome composition of BXD strains. (TXT)

S3 Table Significant QTLs that influence cecum microbial composition in the gut of BXD mice at the genus and species level. A positive additive effect indicates that *DBA/2J* alleles increase trait values. In contrast, a negative additive effect indicates that *C57BL/6J* alleles increase the trait value.
(TXT)
