# Supplementary figures and images for: Diet modulates cecum bacterial diversity and physiological phenotypes across the BXD mouse genetic reference population
Source: PLoS One. 2019 Oct 21;14(10):e0224100. doi: 10.1371/journal.pone.0224100 (PMC6802831; doi:10.1371/journal.pone.0224100)

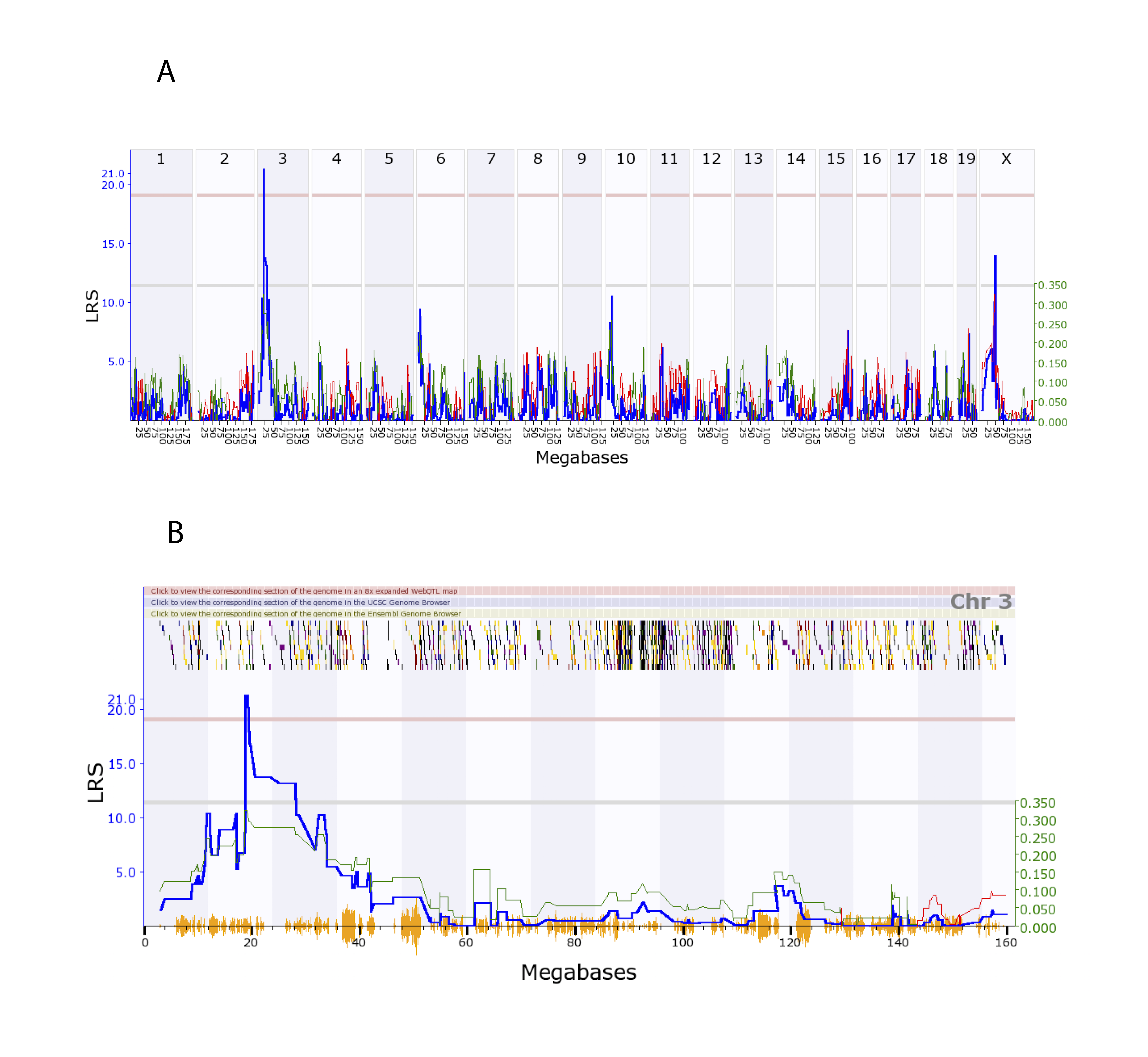

Supplement: S1 Fig — The Left y axis represents the Likelihood Ratio Statistic (LRS) between Oscillibacter composition to different DNA marker intervals on A) each chromosome (blue line) and B) detailed region of the QTL located on Chr 3 (18.7–19.2 Mb) that explains 5.6% of the composition in Oscillibacter. The Right y axis represents the additive effect and indicates if the DBA/2J (green line) or C57BL/6J (red line) alleles contributes to an increase in the abundance of Oscillibacter. Pink and gray horizontal lines indicate the significant (<0.05) and suggestive (<0.67) QTL threshold. (TIFF) [file pone.0224100.s001.tiff]
